# Cell-Type Dependent Effect of Surface-Patterned Microdot Arrays on Neuronal Growth

**DOI:** 10.3389/fnins.2016.00217

**Published:** 2016-05-18

**Authors:** Min Jee Jang, Woon Ryoung Kim, Sunghoon Joo, Jae Ryun Ryu, Eunsoo Lee, Yoonkey Nam, Woong Sun

**Affiliations:** ^1^Department of Bio and Brain Engineering, KAISTDaejeon, South Korea; ^2^Department of Anatomy, Brain Korea 21, Korea University College of MedicineSeoul, South Korea

**Keywords:** microcontact printing, neuron patterning, neuron-surface interaction, neuro-chip design, spinal interneuron

## Abstract

Surface micropatterns have been widely used as chemical cues to control the microenvironment of cultured neurons, particularly for neurobiological assays and neurochip designs. However, the cell-type dependency on the interactions between neurons and underlying micropatterns has been rarely investigated despite the inherent differences in the morphology of neuronal types. In this study, we used surface-printed microdot arrays to investigate the effect of the same micropatterns on the growth of mouse spinal interneuron, mouse hippocampal neurons, and rat hippocampal neurons. While mouse hippocampal neurons showed no significantly different growth on control and patterned substrates, we found the microdot arrays had different effects on early neuronal growth depending on the cell type; spinal interneurons tended to grow faster in length, whereas hippocampal neurons tended to form more axon collateral branches in response to the microdot arrays. Although there was a similar trend in the neurite length and branch number of both neurons changed across the microdot arrays with the expanded range of size and spacing, the dominant responses of each neuron, neurite elongation of mouse spinal interneurons and branching augmentation of rat hippocampal neurons were still preserved. Therefore, our results demonstrate that the same design of micropatterns could cause different neuronal growth results, raising an intriguing issue of considering cell types in neural interface designs.

## Introduction

Micro-contact printing of biologically active materials on the surface of culture substrates has been widely applied to various neurobiological assays and neural interface designs to facilitate control of the extracellular microenvironment *in vitro*. Investigating survival and growth of neurons on patterned substrates is a simple yet highly effective way to unravel the interaction between neurons and extracellular molecules, such as proteins (Chiang et al., 2011; Poudel et al., 2013) or synthetic biomaterials (Liu et al., 2008; Chien and Tsai, 2012). As the height of contact-printed patterns is ~10 nm (Ryu et al., 2016), which exerts a negligible effect on neuronal growth (Baranes et al., 2012), contact-printed patterns were useful to investigate the chemical interaction in neural interface without the topographic effect of surface. Furthermore, the selective neuronal responses to the specific geometry of micropatterns have enabled us to manipulate the initiation and growth of neurites, particularly axons, to the desired direction in nerve regeneration studies, neural tissue engineering, and neural circuit design (Fricke et al., 2011; Jang and Nam, 2012; Hart et al., 2013).

Most of the micropattern-based studies have been executed using long-projecting excitatory neurons, such as hippocampal pyramidal neurons (Liu et al., 2008; Jang and Nam, 2012), cortical neurons (Fricke et al., 2011; Hart et al., 2013), or peripheral ganglion neurons (von Philipsborn et al., 2006). Interestingly, several studies have reported that the responses of neurons to the underlying micropattern differ according to the intrinsic properties of the neuron. For example, VonPhilipsborn et al. showed that nasal retinal neurons grow continuously on ephrin gradient patterns, whereas temporal retinal neurons stop growing at the specific level of the gradient. Therefore, as the morphological characteristics of neurons inherently differ from each other, the same micropattern could have different effects in different types of neurons. Furthermore, different host animals may also affect the results, even if the neurons were isolated from the same region.

In order to investigate the effect of controlled conditions on cell behaviors, an array of microdots has been often employed due to its controllability. A microdot array is easy to realize various microenvironmental conditions with different size and spacing (Chen et al., 1997; von Philipsborn et al., 2006; Fricke et al., 2011). Using different proteins enables controlling the chemical compositions of cellular microenvironment (Ryu et al., 2016). If the patterns become subcellular nanometer scale, it is also possible to control focal adhesion points applicable for mechanotransduction studies (Horzum et al., 2014). It has been reported that the design of microdot arrays determined cell fate in stem cell differentiation (Tonazzini et al., 2013; Yennek et al., 2014; Amin et al., 2016), cell migration (Lehnert et al., 2004; Andersen et al., 2015), and distinct features of cancer cells (Horzum et al., 2015). In case of neuronal cells, patterning various synaptic proteins recruited the counterparts of synaptic proteins in a cultured neuron, thereby facilitating synapse formation assays in the culture condition (Czöndör et al., 2013).

Here we compared the effect of the same micropattern on early growth of several types of neurons in culture. We previously reported the branching initiation effect of microdot arrays using mouse hippocampal neurons (Kim et al., 2014), which also showed that morphological features, such as neurite length and branch number, of mouse hippocampal neurons did not change significantly on the microdot arrays. In this study, however, mouse spinal interneurons (same host animal but different neuronal types) and rat hippocampal neurons (same neuronal type but different host animal) revealed that both neurons reacted differently to the same microdot arrays; unlike the mouse hippocampal neurons, the mouse spinal interneurons extended major neurite longer, whereas the rat hippocampal neurons formed more branches. To clarify whether the distinct responses of the two neuronal types to microdot arrays rely on pattern designs, we diversified the size and spacing of microdot arrays to investigate the changes in the morphological responses of both neuron types on all combinations of microdot arrays. In most cases, the overall growth of neurons tended to be promoted on the denser microdot arrays and restricted on the sparser ones, regardless of neuronal type. However, the specific responses of each neuron, such as early elongation of the major neurite in mouse spinal interneurons and branching augmentation in rat hippocampal neurons, were preserved in the same range of microdot arrays, suggesting the presence of cell-type dependency in the neuron-micropattern interaction.

## Materials and methods

### Fabrication of microdot arrays

The microdot arrays were designed with a combination of three diameters (3, 5, and 10 μm) and four spacings (3, 5, 10, and 20 μm). Spacing indicates the distance between dot edges. A silicon mold was fabricated by photolithography using the SU8-2002 photoresist (Microchem, Woburn, MA, USA). After silanization of the silicon mold with (1H, 1H, 2H, 2H-perfluorooctyl) trichlorosilane (Sigma-Aldrich, St. Louis, MO, USA) for 45 min, the mixture of polydimethoxysilane (PDMS) prepolymer and curing agent (Dow Corning, Corning, NY, USA) was poured into the mold. The PDMS was cured for more than 2 h in an oven (60°C) and was gently released from the mold. Next, it was cut into several pieces (1 × 1 cm^2^) and used as a stamp for the printing procedure.

**Figure 1:**
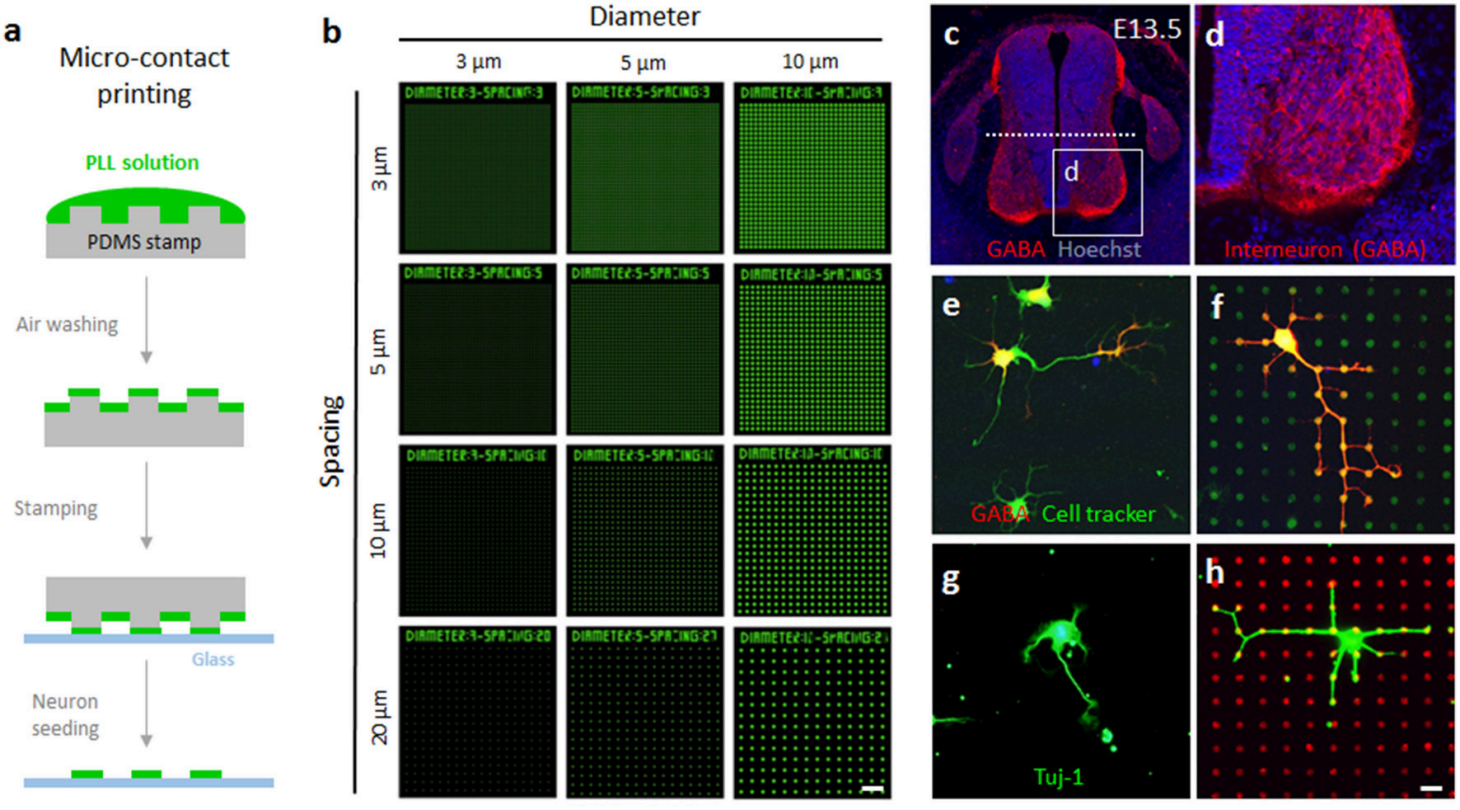
**Fabrication of surface-printed microdot array chips and two different neuron cultures. (A)** Brief procedure to micro-contact printing for fabricating microdot arrays. **(B)** Pseudoimages of the microdot arrays with 3, 5, and10 μm diameters and 3, 5, 10, and 20 μm spacing. **(C,D)** Coronal view of the lumbar spinal cord. The dashed line indicates the middle of the spinal cord to cut for cell culture. **(E,F)** Spinal interneurons expressed GABA (red). Representative images of cultured spinal interneurons co-labeled with cell tracker (green) on the no-pattern substrates **(E)** and microdot arrays **(F). (G,H)** Representative images of cultured rat hippocampal neurons labeled with Tuj-1 (green) on the no-pattern substrates **(G)** and microdot arrays **(H)**. Scale bars in **(B)** and **(C)** indicate 50 and 10 μm, respectively.

A conventional micro-contact printing procedure was employed to print the microdot arrays on the culture substrates (Figure 1A). First, the stamps were sequentially washed with acetone, isopropyl alcohol, and triple-distilled water with ultra-sonication for 5 min each. Then, the stamp was submerged into 10% sodium dodecyl sulfate (Sigma) solution for 15 min. During the first 5 min, the stamps were sonicated to remove bubbles on their surface. Next, a drop of poly-L-lysine conjugated with FITC (0.1 mg/ml in triple-distilled water; Sigma) was loaded on the pattern side of the stamp for 30 min. After the extra solution was removed with compressed air, the pattern side of the stamp was pressed against a coverglass substrate with a 20 g weight. After 1 min of stamping, the coverglass was detached (Figure 1B) and sterilized with 70% ethanol.

### Cell cultures

The ventral column of the lumbar spinal cord from embryonic CD-1 mice at 13.5 days (E13.5) gestation was dissected out (Orient BIO Inc., Seoul, Korea) for the primary mouse spinal cord neuronal cultures. Ventral lumbar tissue was trypsinized for 10 min at 37°C. After the trypsin was inactivated with DMEM containing 10% fetal bovine serum, the lumbar tissue was mechanically dissociated. Following cell filtration with a 40 μm sterile cell strainer (Becton Dickinson, Brea, CA, USA), the dissociated cells were plated at 100 cells/mm^2^ density on polylysine (Sigma)-coated or microdot array-patterned coverslips. Cells were cultured in neurobasal medium containing B27 supplement (Invitrogen, Carlsbad, CA, USA), 0.5 mM L-glutamine, 12.5 μM glutamate, 50 U/ml penicillin-streptomycin (Invitrogen), 20 ng/ml glial cell-derived neurotrophic factor (GDNF), and 200 ng/ml insulin-like growth factor (IGF-1).

Hippocampi of E18 Sprague-Dawley rats were dissected and dissociated in 1 mL HBSS by pipetting for the primary rat hippocampal neuron cultures. The cell suspension was centrifuged for 2 min at 1000 rpm, and the supernatant was gently removed. Plating medium (the same medium for spinal cord cultures except GDNF and IGF-1) was added to re-suspend the cells. The dissociated cells were plated at 100 cells/mm^2^ on substrates, and half of the medium was exchanged with maintenance medium with the same composition of the plating medium except glutamate. All experiments were carried out in accordance with the ethical guidelines and with the approval of the Animal Care and Use Committee of Korea University and KAIST.

### Cell fluorescence labeling

The cell-tracking dye, green CMFDA (5-chloromethyl fluorescein diacetate) cell tracker (1 mM, 1:1000; Invitrogen) was added to the culture media to selectively label living cells. After a 30 min incubation, the tracker containing media was replaced with new media. Cells were fixed with 4% paraformaldehyde (PFA) in PBS for immunocytochemistry at 1, 2, and 3 days *in vitro* (DIV). Gamma-aminobutyric acid (GABA) antibody (1:1000; Millipore Corp., Billerica, MA, USA) was added for 2 h at room temperature to mark the interneurons. After several washes with PBS, Cy3 secondary antibody (Jackson ImmunoResearch Inc., West Grove, PA, USA) was applied for 30 min. Tuj1 antibody (1:500; Abcam, Cambridge, MA, USA) was added for 1 h at 37°C to label the hippocampal neurons, and Alexa Fluor 488 secondary antibody was subsequently applied for 1 h at room temperature.

We performed immunohistochemical analysis to confirm GABA expression in the neuronal populations of spinal cord tissue. Briefly, the spinal cord of E13.5 mice was fixed in 4% PFA overnight at 4°C. After the spinal cord was cryoprotected with 30% sucrose in PBS overnight, it was sectioned to a thickness of 10 μm. GABA antibody was added to the tissue overnight at 4°C. After several washes with PBS, Cy2 secondary antibody (1:1000; Jackson ImmunoResearch) was applied for 30 min. Tissue sections were washed, mounted, and observed with a confocal microscope (Zeiss LSM510; Carl Zeiss, Goettingen, Germany).

### Data measurement and statistical analysis

We measured major neurite length and the number of axonal branches from the fluorescence images. Major neurite length was measured from the longest neurite of each cell. To measure the number of branches, we selected the longest neurite from each cell and counted the branches that were initiated from the neurite. The length of a major neurite and the number of branches was only considered when the longest neurite was approximately two times longer than the diameter of the neuronal cell body.

The length of a major neurite was traced semi-automatically using NeuronJ, an ImageJ plugin (National Institutets of Health, Betheseda, MD, USA). The number of branches was counted manually from the marked major neurite. The Mann-Whitney test or two-way analysis of variance was used to detect difference between neurons cultured on microdot arrays and those on no-patterned substrates as a control. A *p*-value less than 0.05 was considered statistically significant. All analyses were performed with GraphPad Prism software (GraphPad Software Inc., La Jolla, CA, USA), and all values are given as mean and standard error.

## Results

### Mouse spinal interneurons and rat hippocampal neuron cultures on microdot arrays

GABA-positive inhibitory interneurons were isolated from E13.5 mouse spinal cord (Figure 1C). Immunolabeling of GABA showed that these interneurons were enriched in the ventro-medial domain of the spinal cord (Figure 1D). After dissociation from embryonic tissue, the interneurons were easily identified by GABA expression (Figures 1E,F). However, GABA immunoreactivity was not uniformly distributed in an entire neuron. Therefore, to trace the morphology of individual cells, we used a cell tracker that labels the cell membrane without toxicity. In the following experiments, we only considered cells that exhibited both the cell tracker and GABA as spinal interneurons. A spinal cord culture contains a mixed population of cells, but the majority of cell population in a rat hippocampal culture is pyramidal neurons (Kaech and Banker, 2007). Therefore, following the conventional protocol, we cultured rat hippocampal neurons dissociated from E18 embryos and used the neuron-specific marker Tuj-1 to identify cellular morphology (Figures 1G,H). As a result, 97.9% of mouse spinal interneurons among GABA-positive cells and 48.3% of rat hippocampal neurons among adhered cells was included in our analysis. At each time point in which cell morphology was analyzed, all the neurons are not in the same stage of growth, and neurons of stage 1, 2, and 3 are mixed in dissociated culture according to the classification of developmental stages. In the analysis of growth pattern, we included only stage 3 neurons which bear a rapidly growing neurite to become an axon. More than half of the neurons in rat hippocampal neurons were excluded from the analysis because they are stage 1 or 2 neurons.

### Distinct growth patterns of neurons on no-patterned and patterned substrates

**Figure 2:**
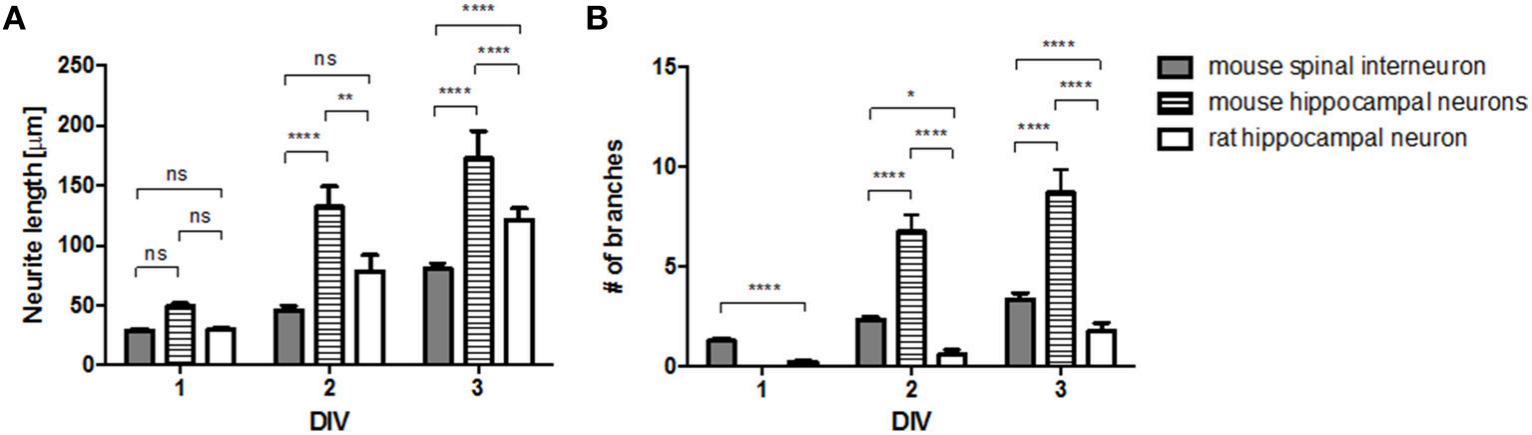
**Cell-type specific growth on no-pattern (control) substrates**. Neurite length **(A)** and branch number **(B)** of mouse spinal interneuron, mouse hippocampal neurons, and rat hippocampal neurons on control substrates (mean ± SEM; ^*^p < 0.05, ^**^p < 0.01, ^****^p < 0.0001 by two-way ANOVA with Bonferroni's multiple comparison test).

Next, we compared the growth profiles of different neurons cultured on the surface of polylysine, which is a uniformly coated adhesive material (Figure 2). We measured the length of neurites (Figure 2A) and the number of branches (Figure 2B) during the first 3 days of culture and found significant differences depending on cell type (hippocampal neuron vs. spinal interneuron) and species (rat vs. mouse). The mouse hippocampal neurons exhibited the most rapid growth with the most branching morphology among three neuronal types. Compared to mouse spinal interneurons, rat hippocampal neurons elongated their neurites faster, whereas they exhibited significantly less branched morphology. These results suggest that the intrinsic growth properties of neurons are different on the same adhesion substrate, indicating the need to evaluate their responses to a micropatterned surface.

**Figure 3:**
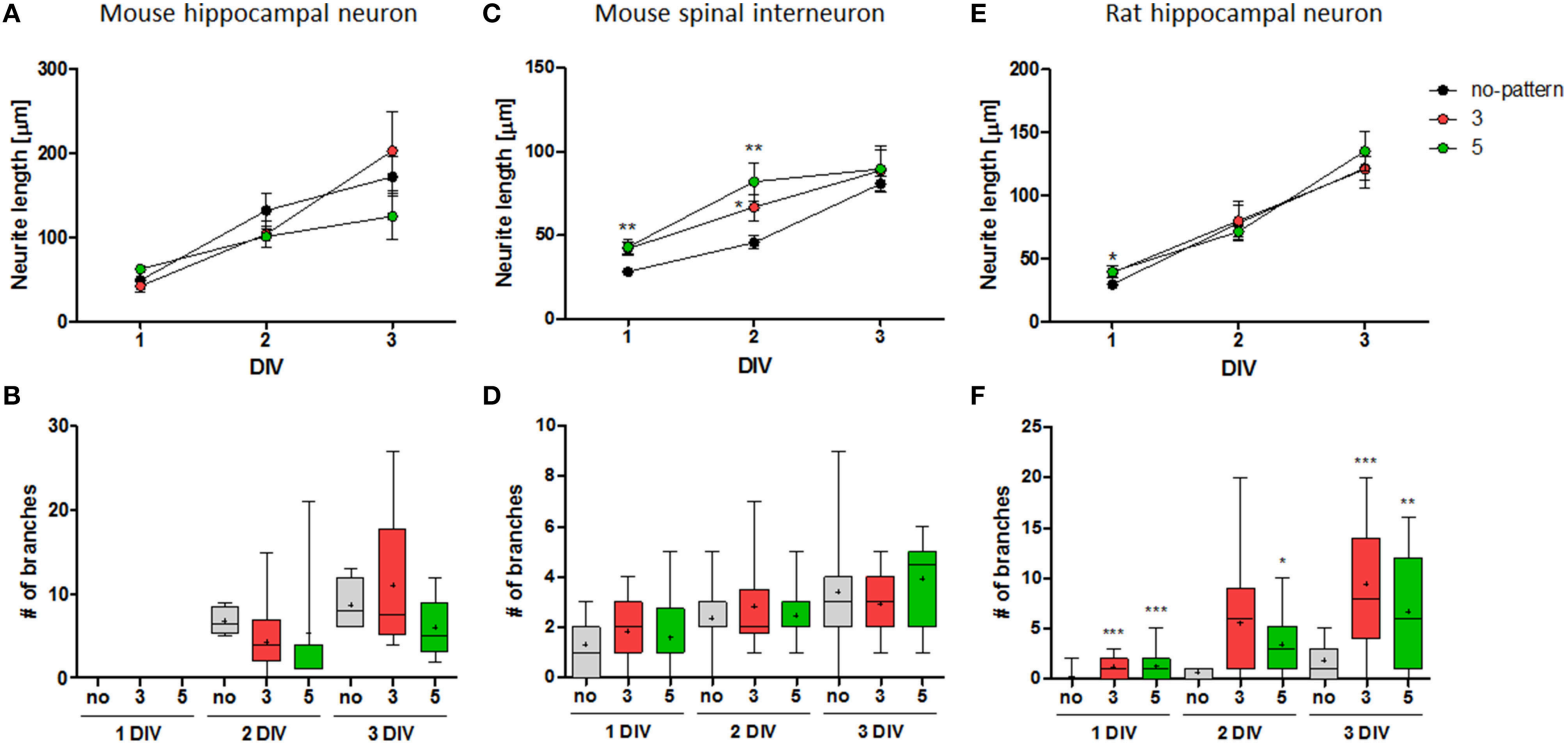
**Cell-type specific growth on microdot arrays**. **(A,B)** The neurite length **(A)** and branch number **(B)** of mouse hippocampal neurons on control substrates (black), microdot arrays with 5 μm diameter and 3 (red) or 5 μm (green) spacings (adapted from Kim et al., 2014). **(C–F)** The neurite length **(C,E)** and branch number **(D,F)** of mouse spinal interneurons **(C,D)** and rat hippocampal neurons **(E,F)** on control substrates and the same conditions of microdot arrays (mean ± SEM; ^*^p < 0.05, ^**^p < 0.01, ^***^p < 0.001 under Mann-Whitney test; **(B,D,F)** box plots with min-max whiskers, +: mean).

Adapted from our previous study (Kim et al., 2014), we compared the growth of mouse spinal interneurons and rat hippocampal neurons with that of mouse hippocampal neurons on the same conditions of microdot arrays (5 μm diameter and 3/5 μm spacing; Figure 3). Unlike mouse hippocampal neurons that had similar neurite length and branch number on patterned substrates at each condition in comparison to the control group (Figures 3A,B), mouse spinal interneurons and rat hippocampal neurons showed significant elongation of major neurite (Figure 3C) and increment of axonal branches (Figure 3F), respectively. The branch number of mouse spinal interneurons and the neurite length of rat hippocampal neurons were not significantly different on microdot arrays (Figures 3D,E).

The results indicate the distinct morphological responses of each neuron on the same micropattern, implying the presence of its cell-type dependency. However, we also observed that the growth of neurites, represented by neurite elongation and branching augmentation, was proceeded at different rates according to the spacing between microdots. For example, the major neurite of mouse spinal interneurons was slightly longer on the microdot array with 5 μm spacing than one with 3 μm spacing, especially at 2 DIV (Figure 3C). Rat hippocampal neurons on the 3 μm spaced microdot arrays showed slightly more branches than on the 5 μm spaced microdot arrays (Figure 3F). It raised two possibilities in our results; first, microdot arrays could have an effect on increasing neurite length or branch number, but the effective range could be cell-specific. Second, the interaction between neurons and microdot arrays could be simply cell-type dependent, regardless of the design parameters. To clarify this issue, we quantitatively traced the growth of mouse spinal interneurons and rat hippocampal neurons on the microdot arrays with various diameters (3, 5, or 10 μm) and spacings (3, 5, 10, or 20 μm) for early 3 days of cultivation.

### Morphological characterization of mouse spinal interneurons grown on microdot arrays

**Figure 4:**
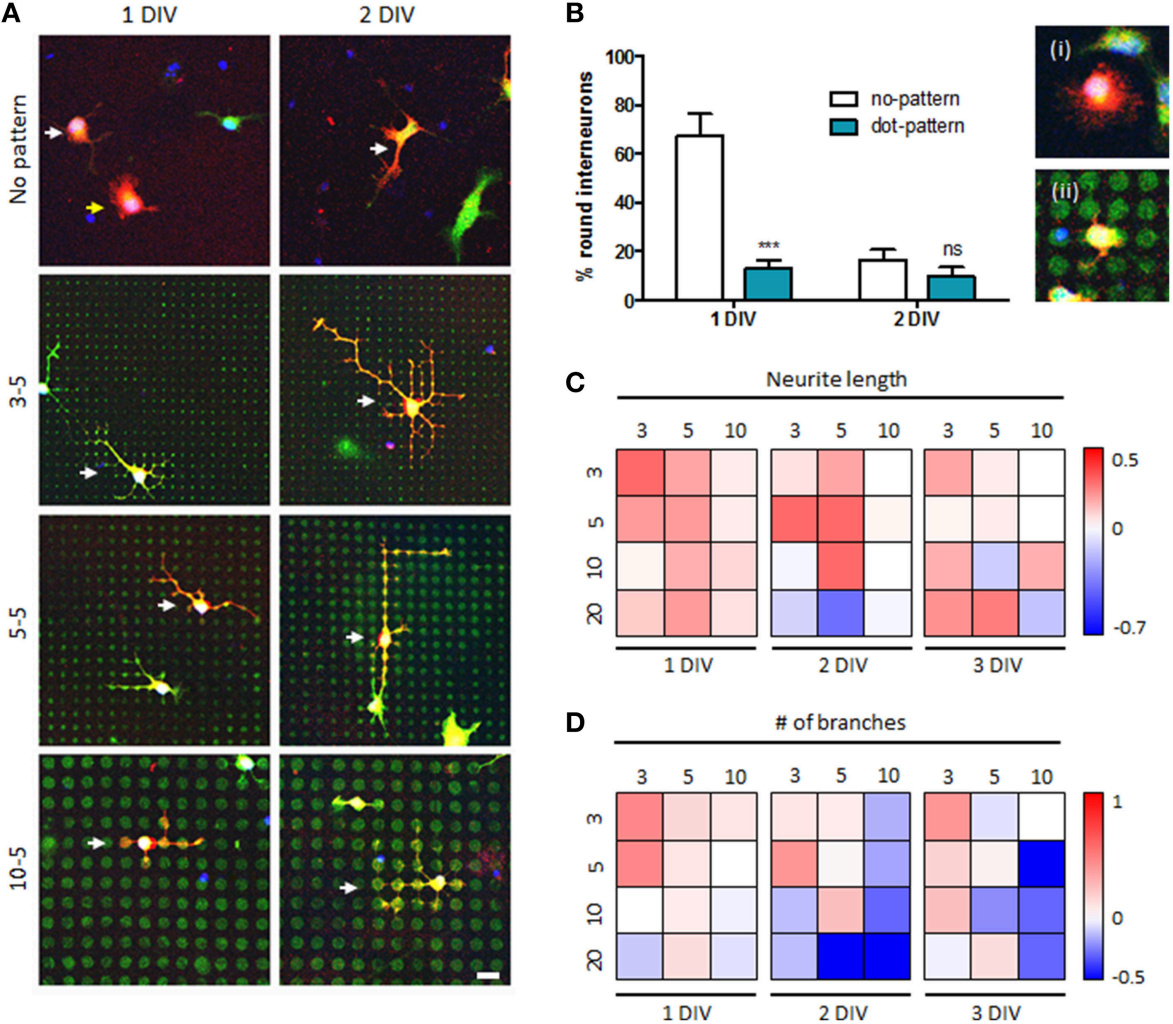
**Accelerated growth of mouse spinal interneurons on microdot arrays**. **(A)** Mouse spinal interneurons cultured on the control substrates or microdot arrays with 3, 5, and 10 μm diameters and 5 μm spacing at 1 or 2 days in *vitro* (DIV). Scale bar indicates 20 μm. **(B)** The ratio of round interneurons on control substrates and microdot arrays (ii; 10 μm diameter and 5 μm spacing; red: GABA, green: cell tracker; mean ± SEM; ^***^p < 0.001, ns: no significant by Mann-Whitney test). **(C,D)** Ratio of neurite length **(C)** and the number of branches **(D)** of mouse spinal interneurons at 1, 2, and 3 DIV compared to the mean value of the control groups under each condition. Each box corresponds to the combination of a diameter (horizontally) and spacing (vertically), and pseudo-colored boxes indicate log ratios.

Next, we investigated growth of mouse spinal interneurons cultured on various microdot arrays (Figure 4 and Supplementary Figure 1). Figure 4 shows the quantitative results of measuring neuronal morphology on the microdot arrays of different diameters. In general, neurons adhered on a surface are initially round and extend thin fibrous neurites over time. Therefore, we measured the ratio of round interneurons, which had not initiated neurites yet, on control (“no-pattern”) and patterned (“dot-pattern”) substrates at 1 and 2 DIV to analyze the initial shape of the cultured neurons. As a result, more than 60% of the neurons were still round at 1 DIV, but this ratio decreased to below 20% at 2 DIV in the control group ((i) in Figure 4B). In contrast, the ratio of round interneurons on the microdot arrays was already below 20% at 1 DIV ((ii) in Figure 4B). The ratio of round interneurons in both groups reached similar level at 2 DIV (<20%; no significant difference). These results indicate that neurite initiation occurred much earlier on the microdot arrays, suggesting accelerated early development of mouse spinal interneurons on the patterned substrate.

To comprehensively investigate the relationship between cell type dependency of the microdot arrays and their designs, we examined the trend in the neuronal morphological responses to changes in the size and spacing of the microdot arrays. Figures 4C,D shows the log ratio of neurite length (Figure 4C) and the number of branches (Figure 4D) compared to those in the control group under each condition. In this figure, the red and blue boxes indicate the positive and negative effects of the microdot arrays, respectively; for example, the most left-top red box in Figure 4C shows that neurite length of mouse spinal interneurons on the 3/3 microdot array at 1 DIV was longer than that in the control group, whereas the white boxes indicate no difference between the microdot arrays and control groups. According to the analysis, there was a tendency for the ratios of the two parameters to be >1 on the microdot arrays with a small dot size and short spacing, regardless of DIV. When the size and spacing of the microdot arrays was increased, the ratio values dropped gradually to the control level (indicated by white boxes) or further decreased to <1 (indicated by blue boxes). In addition, most of the conditions we tested promoted neurite elongation, whereas the same conditions often inhibited branching, suggesting that the microdot array design, such as microdot density, affected the neuronal growth pattern.

### Morphological characterization of rat hippocampal neurons grown on microdot arrays

**Figure 5:**
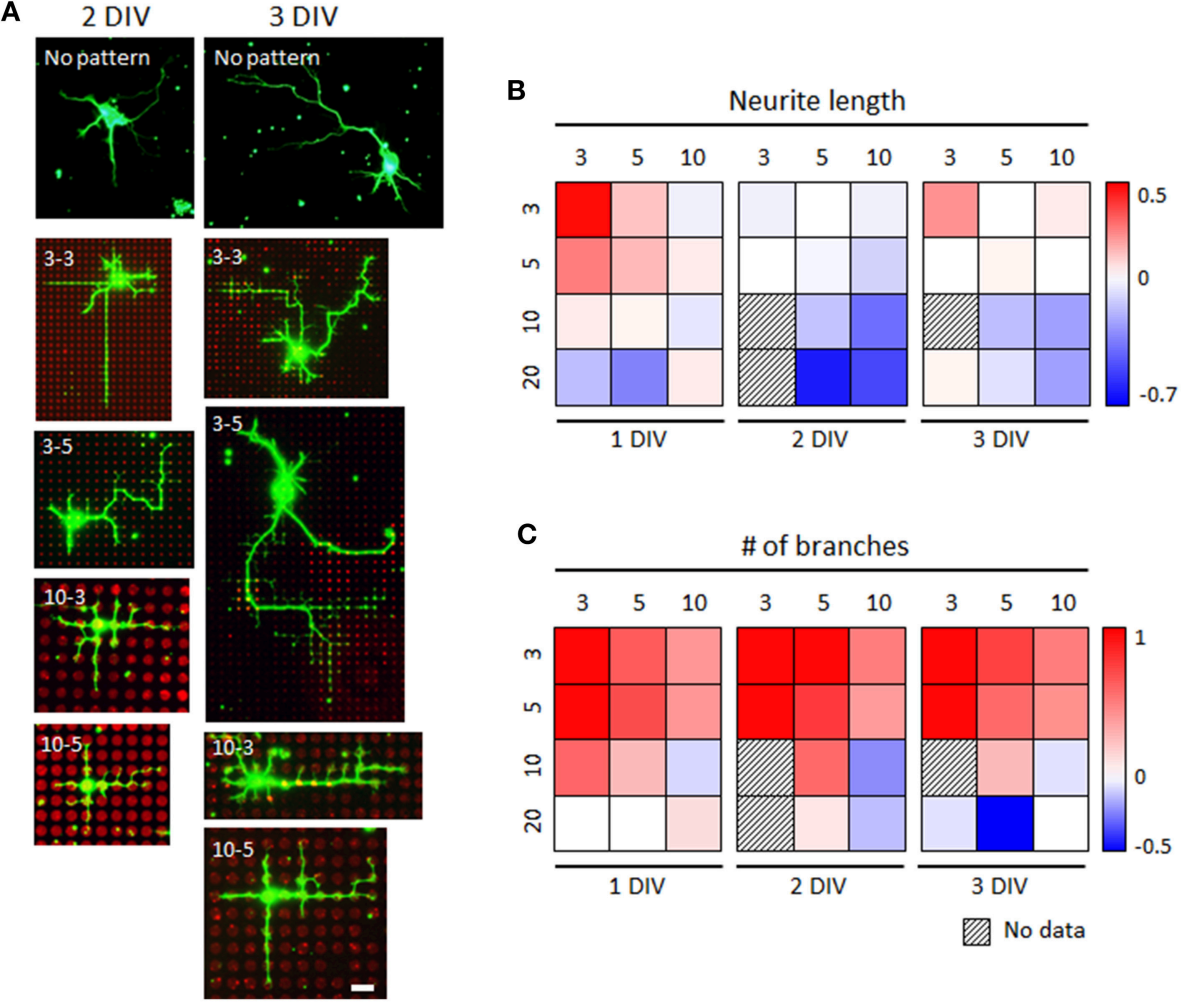
**Branching augmentation of rat hippocampal neurons on the microdot arrays**. **(A)** Representative images of cultured rat hippocampal neurons on control substrates (“No-pattern”) or microdot arrays with 3 or 10 μm diameter and 3 or 5 μm spacing at 2 or 3 DIV (3-3: 3 μm diameter and 3 μm spacing; 3-5: 3 μm diameter and 5 μm spacing; 10-3: 10 μm diameter and 3 μm spacing; and 10-5: 10 μm diameter and 5 μm spacing). **(B,C)** Ratio of neurite length **(B)** and the number of branches **(C)** of rat hippocampal neurons at 1, 2, and 3 DIV compared to the mean value of the control groups under each condition. Each box corresponds to the combination of a diameter (horizontally) and spacing (vertically), and pseudo-colored boxes indicate log ratios.

Next, we investigated the growth of rat hippocampal neurons on the same microdot array designs (Figure 5 and Supplementary Figure 2). We observed a similar tendency for mouse spinal interneurons that the ratios of the two parameters were >1 on the microdot arrays with small dots and short spacing regardless of DIV (Figures 5B,C). When the size and spacing of the microdot arrays increased, the ratios dropped gradually to the control level or further decreased to <1. However, most conditions we tested were rather neutral or inhibitory for neurite elongation of rat hippocampal neurons, whereas the same conditions promoted branching. These results suggest that there was a common neuronal response to microdot arrays, but the precise parameters promoting/inhibiting neuronal growth depended on cell type.

### Common effect of microdot arrays on neuronal branching initiation and adhesion

**Figure 6:**
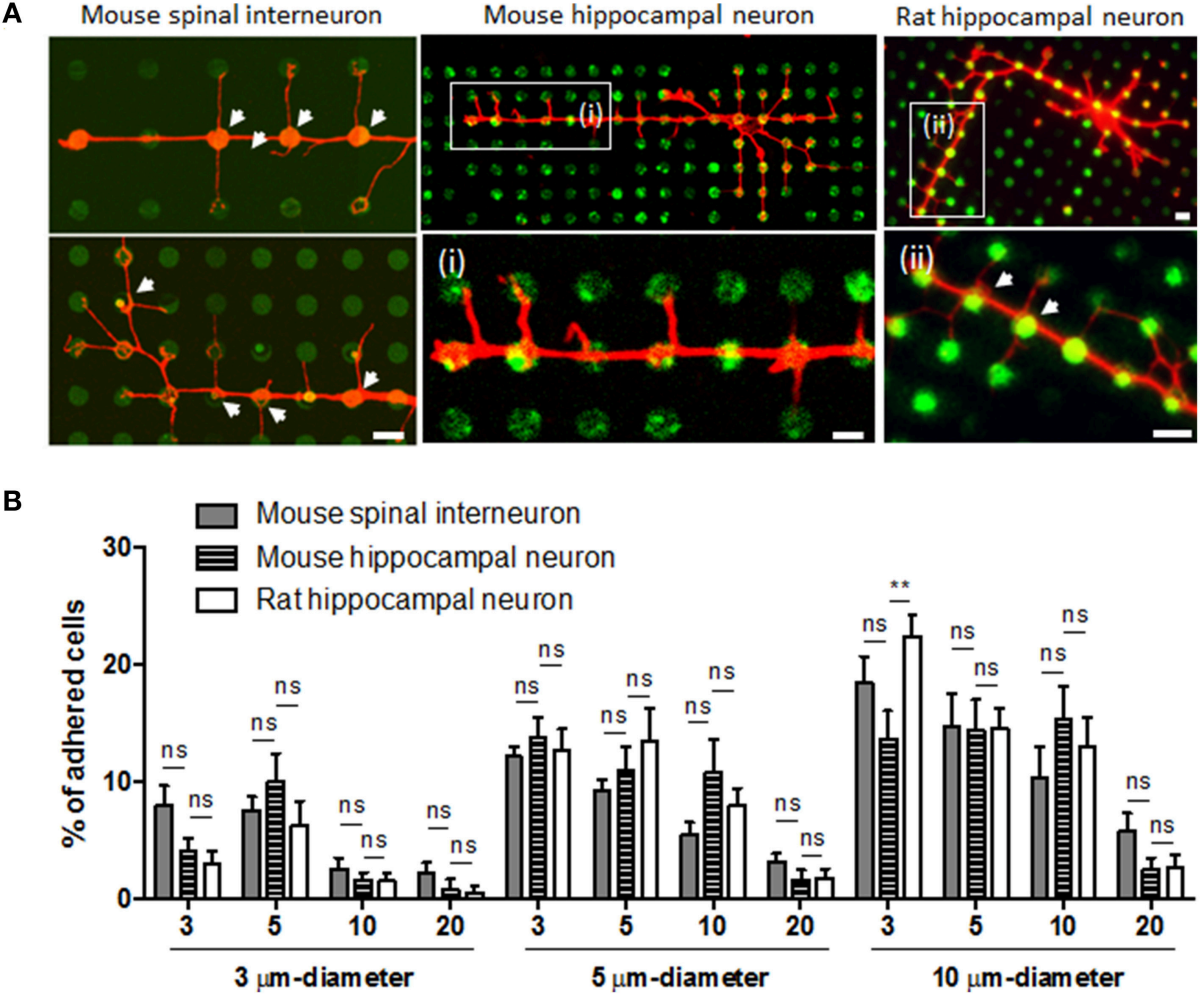
**Branching initiation and neuronal adhesion on the microdot arrays. (A)** The common effect of microdot arrays on localized initiation of axon collateral branches (white arrows) was consistently observed in the spinal interneurons (i, ii; red: GABA) and rat hippocampal neurons (iii, iv; red: Tuj-1). Scale bars in (iv) indicate 10 μm. **(B)** The adhesion efficiency of mouse spinal interneurons, mouse hippocampal neurons, and rat hippocampal neurons on the microdot arrays (mean ± SEM; ^**^p < 0.01, ns: no significance under two-way ANOVA with Dunnett's multiple comparisons test).

Although we found different growth responses depending on neuronal origin and type, all neurons showed similar branching initiation responses from the dots. Regardless of dot size and spacing, all neurons tested exhibited strong constraints on initiating branching from the dots (Figure 6A).

We also examined whether cell adhesion efficiency on various microdot arrays could be varied between neuronal types, as their original size is different. However, the proportion of attached cells on each microdot array was not significantly different between three neuronal types (Figure 6B; two-way ANOVA). Regardless of cell type, there was consistent tendency that larger diameter and shorter spacing resulted in more cells adhering. In further analysis, we found that the ratio of cell population in the mixed culture of mouse spinal interneurons differed between the control and patterned substrates. Approximately 30% of all of these cells were GABA-positive in the control group, whereas most of the cultures on microdot arrays showed a significantly higher ratio of GABA-positive cells (Supplementary Figure 3). Because low percentage of cells was adhered in dot-patterned substrates as shown in Figure 6B, GABA-positive cells might be enriched on the dot-patterned substrates owing to the preferential attachment. However, it should be noted that we monitored cell density 1–3days after seeding, and we are not able to entirely rule out other possibilities such as differential cell survival and differentiation. This result suggests that the total number of adhering cells is based simply on the size of the adhesive area, but that the proportion of cells in the mixed culture varied on the patterned substrates.

## Discussion

In this study, we extensively investigated the morphological responses of early neuronal growth based on microdot array design and neuronal type. Our results suggest that the effects of the microdot arrays on neuronal growth were universally similar between different types of neurons. However, the neuronal types responded differently within a specific range of the microdot arrays. Although mouse hippocampal neurons did not significantly react to the underlying microdot arrays (Kim et al., 2014), spinal interneurons preferred to grow longer. Although the same neuron types were used, hippocampal neurons from rats preferred to form more branches in response to the microdot arrays than those from mice.

The similar trend that neurite length and branch number increased as microdot density increased in both neuronal types suggests that there might be a general rule for the interaction between neuronal growth and the microdot arrays. The fundamental principle of micropatterns as extracellular cues is based on modulation of cytoskeleton structure by making contact with membrane proteins (Théry, 2010; Nam, 2012). Therefore, the effect of microdot arrays could also be applied to both neuron types in the same way, thereby resulting in promoted or depressed growth depending on the design parameters. The same effect of the microdot arrays on cytoskeleton organization was observed with a single microdot. According to our observations, one microdot played a role as an initiation point for axon collateral branching regardless of cell type (Figure 6A). This observation corresponded to our previous report using mouse hippocampal neurons, which showed that a single microdot acts as a localization point of intracellular components for branching (Kim et al., 2014). These results suggest that the local interaction between a single microdot and part of a neuron are consistent regardless of neuronal type. Therefore, the fundamental mechanisms of the neuron-micropattern interactions based on modulation of intracellular cytoskeleton organization seemed to be preserved in the different types of neurons.

Nonetheless, it is particularly interesting that the response of each neuron varied within a specific range of microdot arrays. We noticed that their responsive ranges might be associated with their intrinsic growth characteristics; the neurites of mouse spinal interneurons extended remarkably in the early stage of culture on the microdot arrays (Figures 3C, 4), whereas the rat hippocampal neurons formed more branches (Figures 3F, 5). In contrast, the rat hippocampal neurons under the control condition showed more elongation of a major neurite, whereas the mouse spinal interneurons had more branches (Figure 2). As the growth of a putative axon proceeds by either branching or lengthening, we assumed that these two intrinsic forces guiding neuronal growth might coexist but are biased differently in each neuronal type. According to the comparisons with the two control groups, mouse spinal interneurons seemed to prefer branching, whereas rat hippocampal neurons preferred lengthening (Figure 2). Therefore, the microdot arrays as extrinsic cues appeared to influence the recessive intrinsic tendency more sensitively in both neuron types, whereas the dominant intrinsic tendency was less sensitive to extracellular cues.

**Figure 7:**
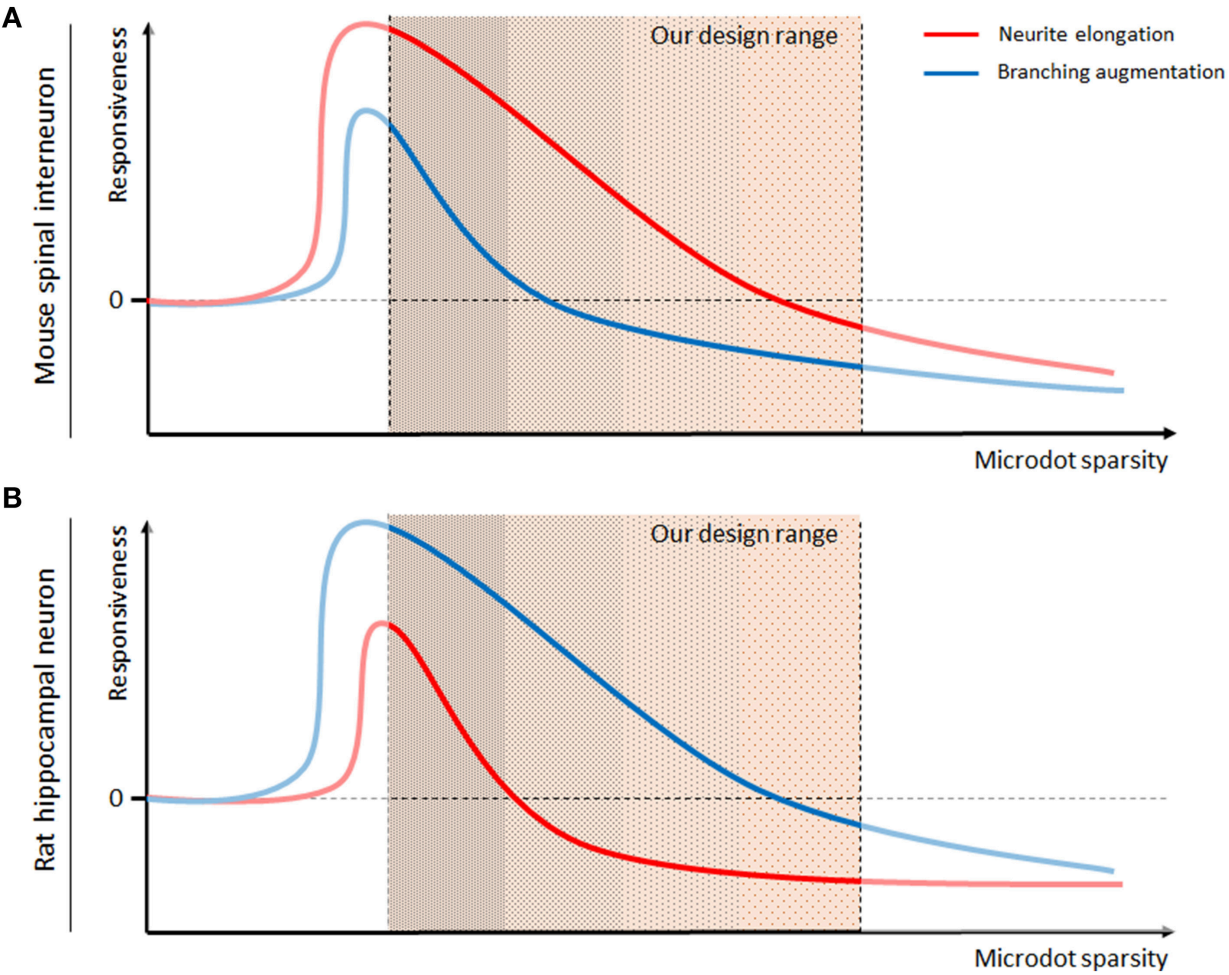
**Model of the cell-type specific interactions between neurons and the microdot arrays**. Responsiveness, defined as the log ratio of neurite length (red lines) or branch number (blue lines) on the microdot arrays to the control groups, of mouse spinal interneurons **(A)** and rat hippocampal neurons **(B)**, was estimated against the sparsity of microdots.

Taken together, we conclude that the responsiveness of neurons to the microdot arrays depended on their type (Figure 7). The responsiveness of neurite length (red) and branch number (blue), which was defined as the log ratio described in Figures 4, 5, was high on the dense microdot arrays but decreased gradually on the sparse pattern. As the extreme case of the dense microdot arrays equates to a no-patterned substrate, the responsiveness would be close to 0 at the initial point of microdot sparsity. This trend was commonly preserved in the mouse spinal interneurons (Figure 7A) and rat hippocampal neurons (Figure 7B), but the more sensitive morphological features of each neuron were different. Mouse spinal interneurons were more sensitive to neurite elongation than branch augmentation, whereas rat hippocampal neurons showed higher sensitivity for branching than elongation. This sparsity-responsiveness relationship also reflects our previous observations about no changes in growth of mouse hippocampal neurons. Considering that mouse hippocampal neurons exhibit remarkably superior growth and branching rates than mouse spinal interneurons and rat hippocampal neurons (Figure 2), we speculate that our microdot array design may be beyond the effective range and that their growth might be affected by the different ranges of the microdot arrays.

Different cellular responses to the same surface pattern were previously reported in several studies (von Philipsborn et al., 2006; Horzum et al., 2015). If the cell was abnormal, like cancer cells, its response to nanodot arrays composed of extracellular matrix proteins was different from that of normal cells (Horzum et al., 2015). When two different types of retinal ganglion cells met the microdot arrays mimicking ephrin gradient, temporal retinal ganglion cells stopped growing at the specific point of the pattern, whereas nasal retinal ganglion cells grew beyond the same point. In the same context, our results consistently showed that the overall growth patterns of different neuronal types varied on the same microdot arrays (von Philipsborn et al., 2006). However, the underlying mechanisms in cell-type dependency of our microdot array might be different from the previous works. In the previous studies, all the patterns were composed of specific proteins that were expected to be effective on the behaviors of each cell. For instance, ephrin was already known to guide axons to the target destination during development (von Philipsborn et al., 2006). Thus, surface patterns could activate the intracellular signaling by binding to the specific receptors in cell membrane, possibly resulting in the different responses regarding to cell type. On the other hand, as we only used synthetic biopolymer (PLL) that has a lot of positive charges for microdot arrays, the major force between the cell and pattern is an electrostatic interaction, suggesting no specificity for triggering intracellular signaling pathways. Therefore, cell-type dependency on neuronal growth in microdot arrays indicates that cell-autonomous differences of growth profiles, such as neurite length and branch number (Figure 2), are sufficient to trigger cell-type dependent responses to the electrostatic surface cues.

## Conclusion

In this work, we showed that surface-printed microdot arrays preferentially induced accelerated early growth of mouse spinal interneurons and a branching morphology of rat hippocampal neurons. Although a similar trend existed in the neurite length and branch number of both neurons in a broad range of microdot arrays, the dominant response of each neuron, neurite elongation of mouse spinal interneurons and branching augmentation of rat hippocampal neurons, still appeared in the specific microdot arrays. The results suggest the presence of optimal microenvironmental features sensitive to neuronal growth as well as distinct morphological responses of neurons depending on their types or species. Thus, our study raises an intriguing issue of cell-type dependency on surface patterns, which has not been extensively considered in neural interface design. This issue will make it possible to design cell-type specific cues to independently manipulate a co-culture system.

## Author contributions

MJJ, WRK, YN, and WS designed this study, discussed the results, and contributed to the writing of the manuscript. MJJ, WRK, JRR, and EL performed the experiments and collected the data. SJ contributed to fabricate micropatterned substrates. MJJ and WRK analyzed the data.

### Conflict of interest statement

The authors declare that the research was conducted in the absence of any commercial or financial relationships that could be construed as a potential conflict of interest.
