# Supplementary figures and images for: Cell-Type Dependent Effect of Surface-Patterned Microdot Arrays on Neuronal Growth
Source: Front Neurosci. 2016 May 18;10:217. doi: 10.3389/fnins.2016.00217 (PMC4870857; doi:10.3389/fnins.2016.00217)

## Slide 1
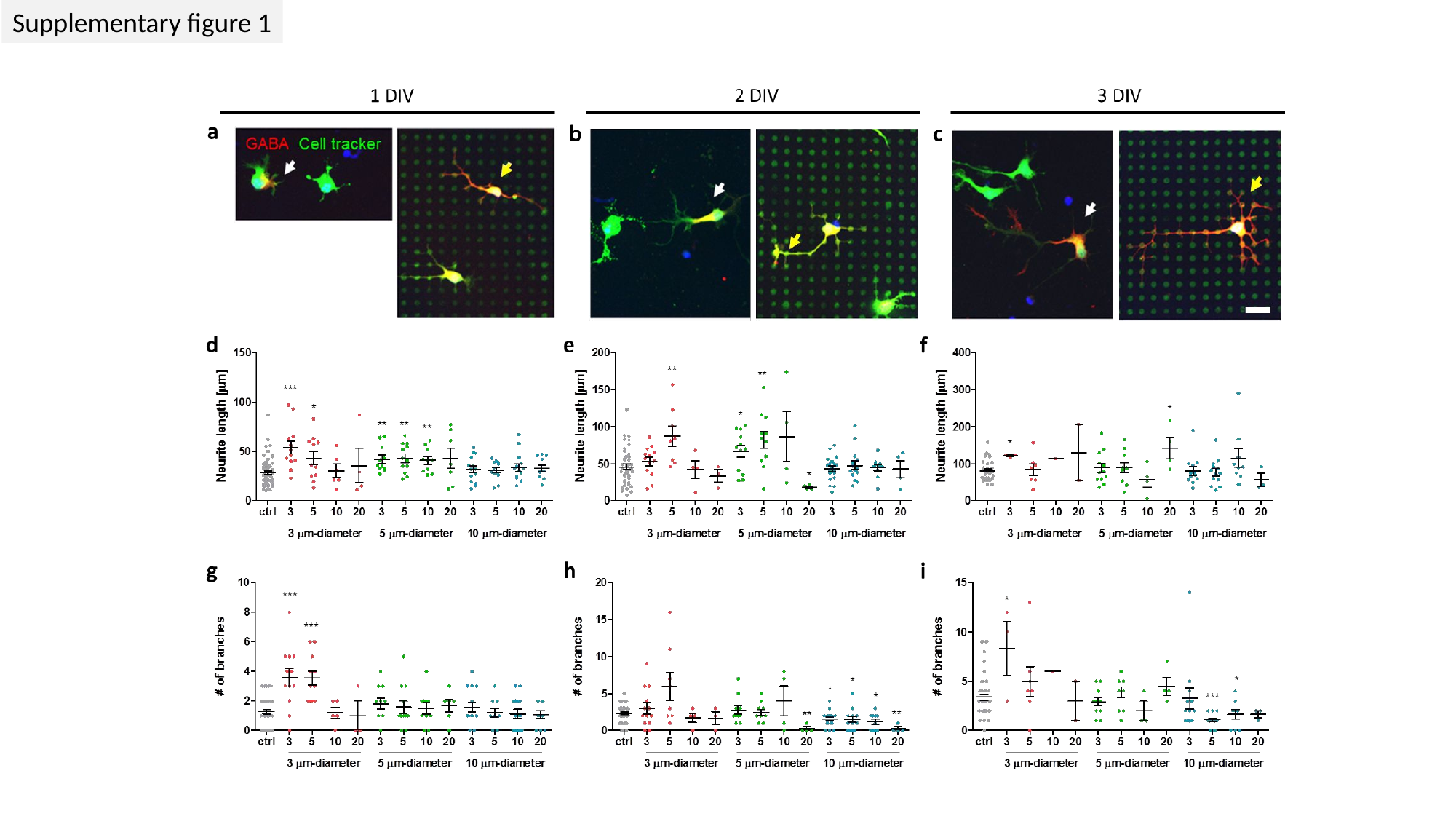

Supplementary figure 1

## Slide 2
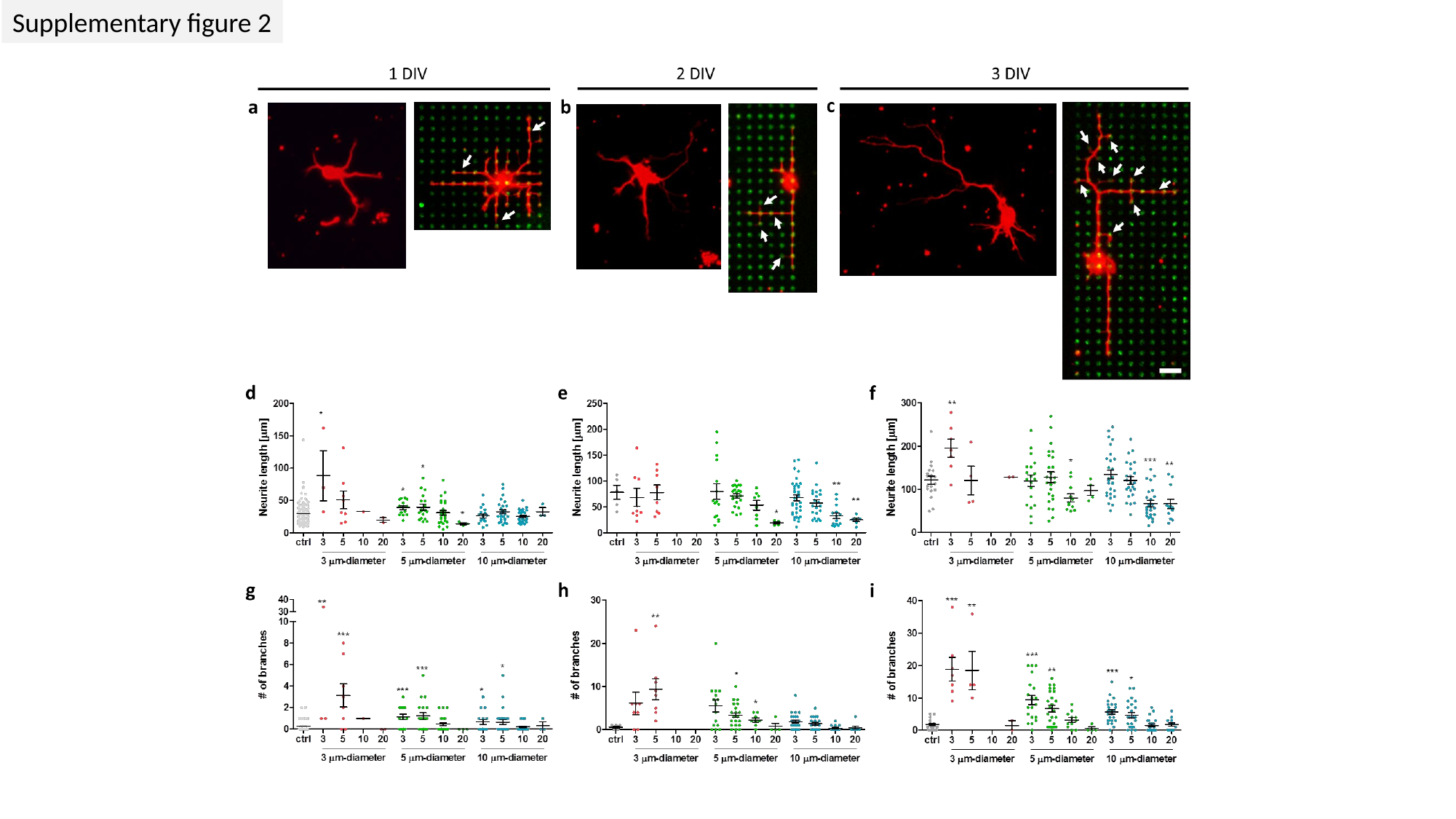

Supplementary figure 2

## Slide 3
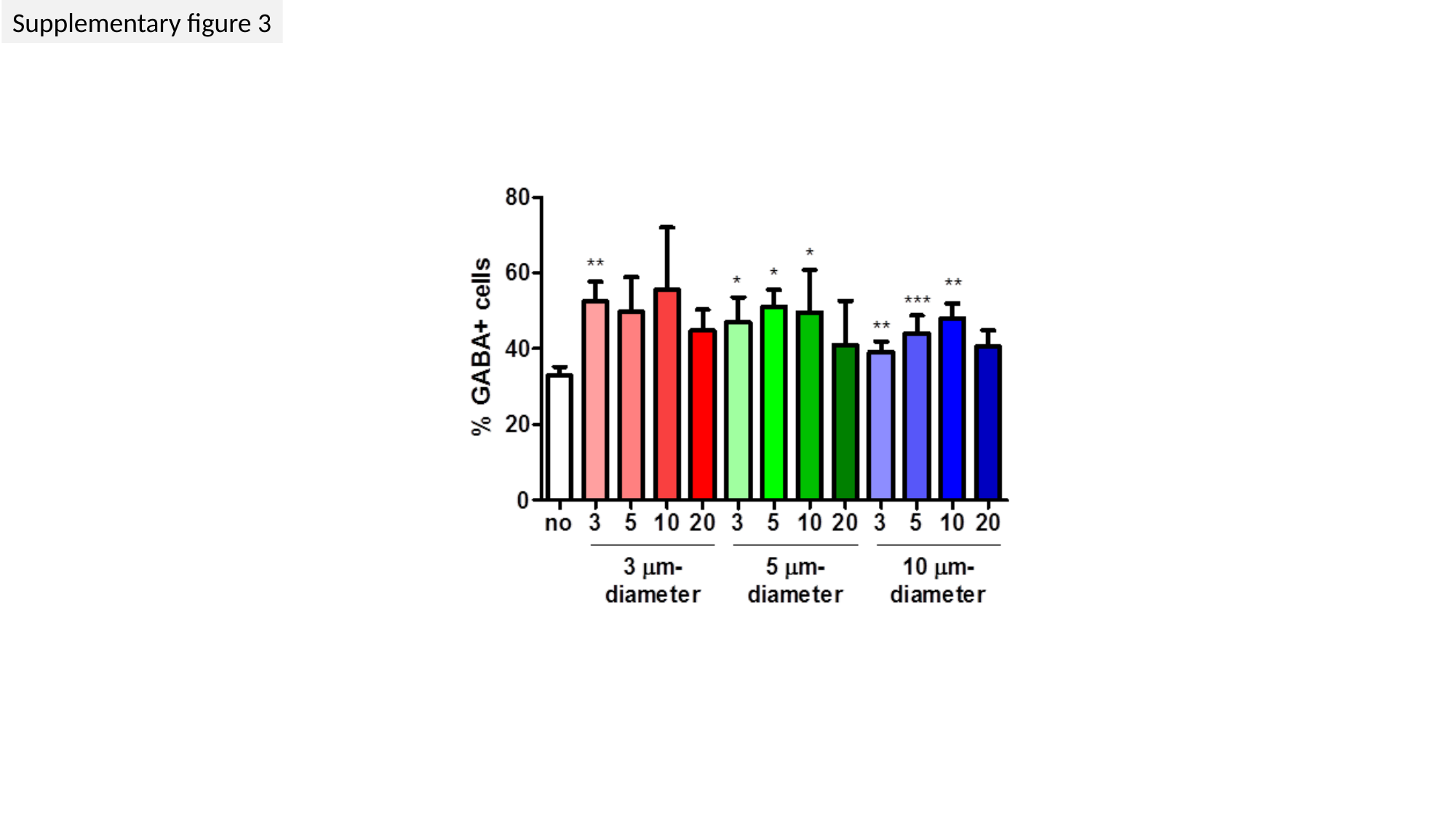

Supplementary figure 3

Supplement: Supplementary file 1 [file Presentation1.PPTX]
